# Protein Misdirection Inside and Outside Motor Neurons in Amyotrophic Lateral Sclerosis (ALS): A Possible Clue for Therapeutic Strategies

**DOI:** 10.3390/ijms12106980

**Published:** 2011-10-19

**Authors:** Akemi Ido, Hidenao Fukuyama, Makoto Urushitani

**Affiliations:** 1 Unit for Neurobiology and Therapeutics, Molecular Neuroscience Research Center, Shiga University of Medical Science, Seta-Tsukinowa-cho, Otsu, Shiga 520-2192, Japan; E-Mail: iakemi@belle.shiga-med.ac.jp; 2 Human Brain Research Center, Kyoto University Graduate School of Medicine, Kyoto, Japan; E-Mail: fukuyama@kuhp.kyoto-u.ac.jp

**Keywords:** seeding, subcellular localization, SOD1, TDP-43, non-cell-autonomous motor neuron death, antibody

## Abstract

Amyotrophic lateral sclerosis (ALS) is a devastating neurodegenerative disease characterized by progressive muscle wasting and weakness with no effective cure. Emerging evidence supports the notion that the abnormal conformations of ALS-linked proteins play a central role in triggering the motor neuron degeneration. In particular, mutant types of superoxide dismutase 1 (SOD1) and TAR DNA binding protein 43kDa (TDP-43) are key molecules involved in the pathogenesis of familial and sporadic ALS, respectively. The commonalities of the two proteins include a propensity to aggregate and acquire detrimental conformations through oligomerization, fragmentation, or post-translational modification that may drive abnormal subcellular localizations. Although SOD1 is a major cytosolic protein, mutated SOD1 has been localized to mitochondria, endoplasmic reticulum, and even the extracellular space. The nuclear exclusion of TDP-43 is a pathological hallmark for ALS, although the pathogenic priority remains elusive. Nevertheless, these abnormal behaviors based on the protein misfolding are believed to induce diverse intracellular and extracellular events that may be tightly linked to non-cell-autonomous motor neuron death. The generation of mutant- or misfolded protein-specific antibodies would help to uncover the distribution and propagation of the ALS-linked proteins, and to design a therapeutic strategy to clear such species. Herein we review the literature regarding the mislocalization of ALS-linked proteins, especially mutant SOD1 and TDP-43 species, and discuss the rationale of molecular targeting strategies including immunotherapy.

## 1. Introduction

Amyotrophic lateral sclerosis (ALS) is a fatal paralytic disease with no effective cure. Although the etiology of ALS remains unclear, the current consensus implicates aberrant conformational changes in disease-associated proteins driving the diverse signaling cascades causing ALS [1–3]. The original pathological evidence came from case reports describing cellular inclusions either reminiscent of Lewy bodies in a familial ALS case [4] or resembling Lafora bodies in a sporadic ALS patient [5]. The Lewy body-like hyaline inclusions as well as the skein-like inclusions, are ubiquitinated [6,7], indicating that ALS pathogenesis involves protein mishandling.

Approximately 90–95% of ALS cases are sporadic with unknown etiology. However, intensive genetic approaches to familial ALS (FALS) patients have successfully identified diverse ALS-causative genes including SOD1, ALS2, SETX, SPG11, FUS, VAPB, ANG, and TDP-43, which are assigned as ALS1, 2, 4, 5, 6, 8, 9, and 10, respectively [8]. Moreover, an increasing number of new genes have been identified in familial ALS including optineurin [9], ubiquilin2 [10], and an expanded GGGGCC hexanucleotide repeat in noncoding region of C9ORF72 [11,12]. Strikingly, the wild type (WT) forms of SOD1, TDP-43, and FUS have been detected in pathological inclusions in sporadic ALS, justifying the research on familial ALS to understand sporadic cases. Indeed, many signaling mechanisms implicated in mutant SOD1-linked ALS may also underlie the pathogenesis of sporadic ALS, including mitochondrial damage, glutamate toxicity, proteasome impairment, ER stress, and axonal flow strangulation [13,14].

Physiological functions of the various ALS-associated proteins may vary widely despite the characteristic motor symptoms of ALS. This indicates huge diversities in ALS pathogenic mechanisms. However, aberrant gene products in ALS, and especially those involved in autosomal dominant inheritance such as SOD1, TDP-43, FUS, and, in several cases, optineurin, have been colocalized with ubiquitin-positive inclusions [9,15–17], indicating the disturbance of clearance pathways of misfolded proteins. Interestingly, the mutant products of these genes show abnormal subcellular distributions in cultured cells and affected tissues. For instance, mutant SOD1 protein, a major cytosolic protein, is mislocalized to mitochondria, endoplasmic reticulum, and the trans-Golgi network, and each such subcellular compartment could be a source of toxic signaling [13]. For example, the redistribution of TDP-43 to the cytosol induces aggregate formation and relocates nuclear TDP-43. Therefore, it is useful and practical to approach ALS pathogenesis from the standpoint of protein mislocalization driven by aberrant conformations.

Here, we review the roles of two major ALS-linked proteins, SOD1 and TDP-43, with a focus on their aberrant subcellular localizations.

## 2. Superoxide Dismutase 1 (SOD1)

### 2.1. Molecular Basis

In 1993, superoxide dismutase 1 (SOD1) was identified as the first genetic mutation in 20% of familial ALS patients [18]. SOD1 is an oxidation antagonist found in the cytosol that scavenges the superoxide to produce oxygen and hydrogen peroxide (O_2_ ^−^ + H_2_O → O_2_ + H_2_O_2_). It is one of the most abundant cellular proteins comprising about 2% of total cytosolic protein content. To date, more than 150 SOD1 mutations have been reported, which together cover most of the functional domains [19]. Moreover, the enzymatic activity of mutant SOD1 varies from null to “increased” despite similar symptoms in overall mutants. Moreover, SOD1 KO mice show no ALS phenotype [20]. These findings indicated that mutant SOD1-linked ALS is caused not by the loss of anti-oxidant function, but by the acquired toxic function resulting from protein conformational change.

Disulfide formation through this Cys57-Cys146 bond stabilizes the SOD1 structure and maintains proper dimerization [21], as shown by the study of Wang *et al*. [22] in which a substitution mutant of cysteine induced the misfolding of SOD1. On the other hand, Cys6 and Cys111 have been implicated in oligomer formation under oxidative conditions [22–24]. Considering that the cytosol is a reducing environment that would result in cleavage of disulfide bonds, this intramolecular Cys57-Cys146 bridge is protected from reduction by the proper dimer conformation. Therefore, it is reasonable to assume that a mutation-derived single amino acid replacement might affect the protein dimer structure, leading to misfolding, particularly as many mutant SOD1s are readily monomerized under mild reducing condition [25]. Rakhit *et al*. [26] reported that both WT and mutant SOD1 transiently form unnatural and partially folded monomers upon oxidation, before oligomerizing or aggregating. Based on these findings, the group then generated a rabbit polyclonal antibody against the potential dimer interface (SEDI), and demonstrated that this antibody specifically labels misfolded SOD1 [27]. We provided further evidence for the significant role of a transient hydrophobic surface by identifying Hsp70-SOD1 complex, which is present in the detergent-soluble, but not in the detergent-insoluble fraction. Moreover, demetallating SOD1 augmented the affinity of Hsp/Hsc 70 for SOD1 in both mutant and WT protein [28]. Mammalian cells precisely recognize misfolded SOD1 species and direct them for degradation through the ubiquitin-proteasome pathway [29]. Therefore, mutant SOD1 has a shorter half-life than the WT protein; however, the degradation capability of proteasome is gradually impaired under the continued expression of mutant SOD1, leading to the accumulation of ubiquitin-positive SOD1 species [29].

### 2.2. Toxic Cascades Caused by Mutant SOD1

SOD1 is a major cytosolic protein. Live-cell imaging of EGFP-fused SOD1 showed a homogenous distribution in the cytosol for both WT and mutant SOD1s. However, mild membrane perforation using digitonin in the presence of ATP to wash out soluble cytosolic protein unveiled punctate or vesicular locations of SOD1 including mitochondria, lysosomes, ER, and trans-Golgi network, and the effect was more apparent with the mutant SOD1 than with WT [30].

**Figure 1:**
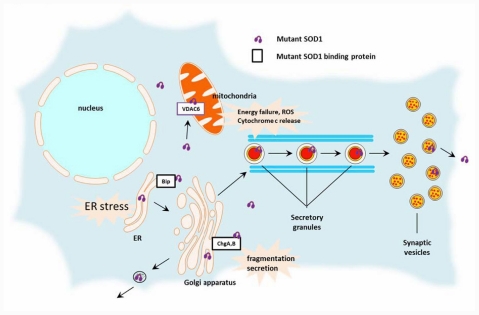
Aberrant subcellular localization of mutant SOD1 protein and the associated effect on ALS pathogenesis. Mutant SOD1 interacts with several “accompanying proteins”, resulting in the abnormal subcellular localization.

Although the molecular basis for these aberrant translocations of mutant SOD1 to various cellular organelles remains elusive, the hydrophobic nature of the mutant protein is a possible explanation. Okado-Matsumoto and Fridovich [31] documented that the apo state of the SOD1 protein enables it to access mitochondria, but the heat shock chaperone prevents entry, indicating that the hydrophobicity of SOD1 is a key factor for its translocation to mitochondria [31]. Apo SOD1 can also be directly incorporated into microsomes in the presence of ATP *in vitro* [30]. Although WT SOD1 has also been localized at lysosomes, mitochondria, and nucleus as well as in the cytosol [32], it should be noted that only the mutant SOD1 causes undesirable detrimental effects on cell survival when redistributed to these other locations (Figure 1).

#### 2.2.1. Mitochondria

Mitochondria are indispensable suppliers of oxidative energy in addition to their role as calcium (Ca^2+^) buffers. In neurons, mitochondria are transported to the distal axon, thus mitochondrial damage seriously affects the diverse functions of the growth cone and synaptic terminals. Indeed, blocking Ca^2+^ entry into mitochondria rescues cultured motor neurons from glutamate-induced cell death [33]. The role of mitochondria in mutant SOD1-linked ALS was first studied using a transgenic approach by Wong *et al*. [34], who demonstrated a prominent vacuolar change to mitochondria in G37R SOD1 Tg mice. Later work revealed mutant SOD1 inside mitochondria in mutant SOD1 Tg mice by immunofluorescence and immunoelectron microscopy [35]. Distinct from WT SOD1, mutant SOD1 induces morphological change and cytochrome c release in cultured neurons that resulted in apoptosis [36]. Two transgenic studies further indicated the involvement of mitochondria-mediated apoptosis in mutant SOD1-linked ALS. Inoue *et al*. [37] demonstrated that suppressing caspase-9 by overexpressing XIAP in motor neurons effectively slowed the progression of ALS in G93A SOD1 Tg mice, while Reyes *et al*. documented that neuron-specific deletion of BCL-associated X protein (BAX) or BCL2-homologous antagonist/killer (BAK), which are both proapoptotic BCL-2 family proteins, delayed the onset and extended the longevity of disease in the same mice [38]. In addition, Vande Velde *et al*. [39] used sophisticated double transgenic mice co-expressing mutant SOD1 and a mitochondria-targeted fluorescent indicator to demonstrate aberrant morphology and distribution of mitochondria. Interestingly, these altered properties were influenced by the dismutase activity of SOD1, whereby dismutase-active G37R SOD1 induces mitochondrial clustering in the proximal site, whereas dismutase-inactive G85R SOD1 produces aberrantly elongated mitochondria in the axon, indicating that oxidative stress modifies the detrimental effect of mutant SOD1 [39]. One of the proposed molecules responsible for the entry of mutant SOD1 into mitochondria is a voltage-dependent anion channel (VDAC1), which resides in the mitochondrial outer membrane [40]. Mutant, but not WT SOD1, interacts with VDAC1, and the ablation of VDAC1 in G37R SOD1 Tg mice slowed the onset of paralysis. Mitochondria are the major source of reactive oxygen species (ROS) in cells, and these could contribute to the observed motor neuron death. However, conditional knockout of SOD2, a mitochondrial SOD, in the motor neurons produced no ALS phenotype [41], arguing against the hypothesis that mitochondrial ROS are a trigger for ALS. Surprisingly, a recent study by Zhu and Sheng [42] revealed that mutant SOD1 Tg mice with increased mitochondrial mobility in the motor neuron axons displayed a similar phenotype to those with impaired mobility. This result suggested that mislocalization of mitochondria alone is not enough to explain the detrimental events caused by the axonal transport impairment, and the malfunction of mitochondria themselves may be directly responsible for the ALS pathogenesis.

#### 2.2.2. Endoplasmic Reticulum (ER)

Previously, the only luminal structures to which the SOD1 redistributed were regarded to be lysosomes and mitochondria [43]. Only one paper using hepatocytes had shown SOD1 in the ER [44]. However, Tobisawa *et al*. [45] showed that mutant SOD1, but not WT protein, colocalized with ER chaperone protein, GRP78/Bip, and induced ER stress in the transfected cells. Studies by our group [46] and by Kikuchi *et al*. [47] used immunoelectron microscopy to more precisely localize the mutant SOD1 in ER. Kikuchi *et al*. also demonstrated that mutant SOD1 interacts with GRP78/Bip, which mediates its entry into ER. We further confirmed the ER localization of SOD1 by sucrose density-gradient ultracentrifugation and floating ultracentrifugation using an iodixanol cushion and high-salt wash [30], while a recent paper demonstrated both WT and mutant SOD1 in microsome fractions and partial colocalization of these proteins with Derlin-1 in ER by immunocytochemistry [48].

ER stress is triggered by the accumulation of unfolded protein in the ER through the activation of three sensor proteins, IRE1, ATF6, and PERK, which downregulate protein synthesis and up-regulate BiP/GRP78 [49]. ER stress is thought to play a crucial role in both sporadic and familial ALS pathogenesis [50–54]. The ablation of BH3-only protein puma, which is necessary to induce ER stress, significantly delayed the disease onset and protected motor neurons [55]. However, the molecular mechanisms by which mutant SOD1 induces ER stress remains unclear. Notably, ER-targeted overexpression of mutant SOD1 did not injure Neuro2a cells [36], suggesting the existence of partner molecules inside or adjacent to ER that are involved in mutant SOD1-induced ER stress. To this end, Nishitoh *et al*. [56] reported that cytosolic mutant SOD1 induced ER stress through an interaction with Derlin-1, leading to an impaired unfolded stress response and the activation of apoptosis signal-regulating kinase 1 (ASK1). In addition, protein disulfide isomerase, which is upregulated under ER stress, shows therapeutic potential based on its amelioration of cell toxicity induced by the unfolded stress response [57].

#### 2.2.3. Golgi Apparatus, Secretory Pathway

Another noxious effect of ER stress caused by mutant SOD1 is fragmentation of the Golgi apparatus, which affects protein maturation and intracellular trafficking of membrane and secretory proteins [58]. Indeed, Gonatas and coworkers [59] clearly showed a fragmented Golgi apparatus in the motor neurons of ALS patients. This does not necessarily mean that mutant SOD1 is present in the Golgi apparatus, since Golgi morphology is affected by the structural dynamism of microtubules for axoplasmic transport. Indeed, SOD1 is synthesized in free ribosomes, and has no signal peptide that drives the entry of proteins into the ER-Golgi secretory pathway. Therefore, despite early findings of WT SOD1 secreted from cultured astrocytes and thymus-derived epithelial and fibroblast cells, secretion of SOD1 is thought to follow a non-classical pathway since brefeldin A, an ER-Golgi transport blocker, did not inhibit the secretion [60].

More recently, Turner *et al*. [61] presented compelling evidence that both WT and mutant SOD1 are trafficked via the classical secretory pathway. In addition to morphological evidence using immunoelectron microscopy, we confirmed the presence of mutant SOD1 in trans-Golgi network of mutant SOD1 Tg mice by immunoisolating the organelle using a Golgi-resident protein, TGN38 [46]. The molecular mechanism used by mutant SOD1 to enter the secretory pathway remains elusive. We identified chromogranin A (ChgA) and B (ChgB), which are neurosecretory proteins, as interacting partners with mutant SOD1, but not WT. Mutant SOD1 and chromogranins interact and colocalize in the G37R and G93A SOD1 transgenic mice, as verified by double immunoelectron microscopy and immunoprecipitation [46]. In addition, both ChgA and ChgB promote the secretion of mutant SOD1, but not WT protein, in cell culture experiments. The potential effects of this secreted and extracellular SOD1 on ALS pathogenesis are discussed below.

### 2.3. Cell-Autonomous and Non-Cell-Autonomous Motor Neuron Degeneration

The overexpression of mutant SOD1, but not WT, induces apoptotic cell death [36,62,63], with mutant SOD1 inducing diverse toxic signaling pathways inside cells, including those driving mitochondrial impairment, ER stress, oxidative stress, and proteasome suppression. However, an earlier study showed that transgenic mice expressing mutant SOD1 predominantly in the motor neuron do not develop ALS [64], while Lino and coworkers [65] showed that mutant SOD1 expression driven by the Thy-1 promoter neither produced an ALS phenotype nor affected the progression of mutant SOD1 Tg mice. On the other hand, transgenic mice harboring GFAP promoter-driven mutant SOD1 showed no palsy, but did show massive astrogliosis [66]. The interplay between motor neurons and glial cells has been intensively investigated by Cleveland’s laboratory. First, Clement *et al*. [67] generated chimeric transgenic mice by randomly mixing morula from mutant SOD1 Tg and control mice (NF-L Tg mice or YFP mice). Their analysis revealed that the number of surrounding cells expressing mutant SOD1 regulates the survival of motor neurons. Specifically, mutant SOD1-expressing motor neurons were not injured if the surrounding cells were non-transgenic. Further studies on the role of motor neurons and glial cells in disease onset and progression, using Cre-loxP system showed that cell-specific downregulation of G37R mutant SOD1 in motor neurons or microglia delays the development or progression, respectively [67]. Yamanaka *et al*. [68] further showed mutant SOD1 in astrocytes plays a crucial role in slowing the progression. This series of studies established the notion that mutant SOD1-induced ALS is mediated by non-cell-autonomous machinery. It should be noted that the type of mutation and promoter used in the experiments would influence these phenomena. Wang *et al*. then showed that depleting the G85R SOD1 mutant in astrocytes delayed the onset and slowed progression of the early symptomatic phase of ALS [69]. This group also documented that restricted expression of the G93A mutant in motor neurons and inter-neurons caused a mild clinical and pathological phenotype [70].

Two independent laboratories recently reported that mutant SOD1-expressing astrocytes secrete molecules that are toxic only to motor neurons [71,72]. One group identified several candidates for these molecules including prostaglandin D2 (PGD2) receptor, Mcp2, Cxcl7, and Rantes [73]. However, a PGD2 inhibitor only partial rescued the function of motor neurons cultured on monolayers of the mutant SOD1-expressing astrocytes; these cells also activate NOX2 to produce superoxide, which may be involved in motor neuron death [73]. Finally, mutant SOD1-expressing microglia were found to produce higher superoxide and nitrite, but lower IGF-1, than WT microglia, and these effects were augmented by LPS stimulation [74].

### 2.4. The Role of Extracellular SOD1 Mutants in the Non-Cell-Autonomous Pathology of ALS

The findings on this topic can be summarized as follows:

1. The density of mutant SOD1-expressing cells in the anterior horns governs motor neuron survival;
2. Mutant SOD1 in motor neurons or glial cells does indeed induce intracellular damage, and the motor neuron degeneration is accomplished by summation of these phenomena;
3. Most of the chemical mediators identified so far are reactive oxygen species or proinflammatory molecules.

Based on our discovery of neurosecretory protein chromogranins as binding partners for mutant SOD1, we hypothesized that mutant SOD1 itself is a mediator of intercellular crosstalk in non-cell-autonomous motor neuron death. Indeed, mutant SOD1 secreted into the culture media, or externally applied recombinant mutant SOD1 protein, activates microglial cell lines and induces motor neuron death in culture [46]. We also documented that the toxicity of extracellular SOD1 mutant is mediated by CD14 on the microglia [75]. Moreover, overexpression of ChgA in G37R SOD1 Tg mice accelerated the disease onset and gliosis [76], while misfolded SOD1 was detected in the cerebrospinal fluid of patients with familial ALS using antibodies specifically recognizing misfolded SOD1 species [77,78].

### 2.5. WT SOD1

Interestingly, even WT SOD1 is misfolded by un- or de-metallation, and is recognized by Hsc70 *in vitro* [28]. Moreover, oxidation of WT SOD1 by H_2_O_2_ both *in vitro* and *in vivo* induces its misfolding and confers toxic effects on cultured motor neurons as well [79]. The role of WT SOD1 in sporadic ALS is a matter of debate. Liu *et al*. [77] failed to detect misfolded SOD1 species in the lumbar spinal cord tissues of sporadic ALS patients using the SEDI antibody, while mutant SOD1 was immunoprecipitated by the same antibody from SOD1-linked familial ALS patients [77]. On the other hand, some work has suggested the involvement of SOD1 in the pathogenesis of sporadic cases as well. Gruzman *et al*. [80] showed that aberrantly dimerized SOD1 is a common finding in spinal cord lysates from sporadic and mutant SOD1-linked familial ALS patients, and recently, motor neurons of sporadic ALS patients were labeled by the monoclonal antibody (C4F6), which recognizes misfolded SOD1 protein, but not natively folded WT SOD1 [81]. Strikingly, Haidet-Phillips *et al*. [82] documented using iPS cells-derived astrocytes from ALS patients, that SOD1 is a target in the toxic machinery in astrocytes in familial and sporadic ALS. The misfolded SOD1 is indeed detected in the CSF of sporadic ALS patients using the antibody against misfolded SOD1, although its concentration is not high enough to induce motor neuron death [78]. However, posttranslational modifications, including oligomerization of a small fraction of SOD1 protein, would enhance the pathogenicity.

## 3. TAR DNA Binding Protein-43kDa (TDP-43)

In 2006, a groundbreaking discovery came from two independent research groups. A nuclear protein, TDP-43, was identified as a core protein of ubiquitinated inclusions of frontotemporal lobar degeneration with ubiquitinated inclusions (FTLD-U) and ALS [83,84]. Genetic mutations in TDP-43 have been detected in a fraction of familial ALS patients with or without FTLD, with several exceptions including pure FTLD [85], FTLD with progressive supranuclear palsy and chorea [86], and FTLD with Parkinsonism [87]. These lines of evidence indicate a primary role for TDP-43 in the development of ALS.

TDP-43 is a DNA/RNA binding protein, the physiological functions of which have been increasingly uncovered, including splicing or stabilization of RNA. The exon 9 skipping of the cystic fibrosis transmembrane receptor (CFTR) is a splicing example of TDP-43 [88]. In relevance to ALS, neurofilament light chain (NFL) mRNA was the first candidate target RNA to be recognized as stabilized by TDP-43 [89]. Recent attempts to grossly screen TDP-43-associated mRNAs using cross-linking and immunoprecipitation (CLIP) from brains of FTLD patients [90], mice brain with or without ablation of TDP-43 by siRNA [91], and human neuroblastoma cell line SHSY-5Y [92] revealed that huge amounts of RNA that bind TDP-43, which interacts with introns at UG/TG-rich repeat motifs of various mRNAs associated with neuronal development and neurological diseases, including TDP-43 itself and progranulin [90–92]. Knocking down TDP-43 in HeLa cells induced apoptosis via the upregulation of cyclin-dependent kinase 6 (cdk6) [93], while TDP-43 deletion in *Caenorhabditis elegans* downregulates histone deacetylase 6 (HDAC6), causing aggregate formation and promotion of the cytotoxicity caused by polyglutamine-expanded ataxin-3 [94]. TDP-43 knockdown in neuronal cultures also inactivated Rho family GTPases, including RhoA, Rac1, and Cdc42, leading to the inhibition of neurite outgrowth and cell death [95]. Based on these data, the current consensus on TDP-43-linked ALS pathogenesis argues for protein misfolding and defective RNA processing [96].

### 3.1. Cytosolic Redistribution of TDP-43 in ALS Pathogenesis

The original and consensus findings for TDP-43 pathologies include the aberrant cytosolic redistribution and the ubiquitinated and phosphorylated inclusions [83,84]. We have shown that both WT and mutant TDP-43 are constitutively polyubiquitinated, and are degraded in proteasomes and autophagosomes [97]. TDP-43 pathology is rarely detected in mutant SOD1-linked ALS, raising the question as to whether these two diseases are essentially different [98]. However, accumulating evidence indicates that long-lived mutant SOD1 Tg mice and a fraction of familial ALS patients with mutant SOD1 show TDP-43 pathology [99]. We also reported a familial ALS patient, with an I112T mutation in SOD1, presenting with massive expression of phosphorylated TDP-43 in the motor neurons of the brainstem and cervical cord [100]. Notably, TDP-43 redistribution has also been reported observed in several conditions not related to ALS or FTLD, including Perry syndrome [101], Lewy-body disease [102], Huntington disease [103], and inclusion body myositis [104]. Axonal injury also induces a transient redistribution of TDP-43 in rodent motor neurons [105,106].

### 3.2. Nuclear Localizing Signal (NLS) and the Responsible Domains for Cytosolic Redistribution

**Figure 2:**
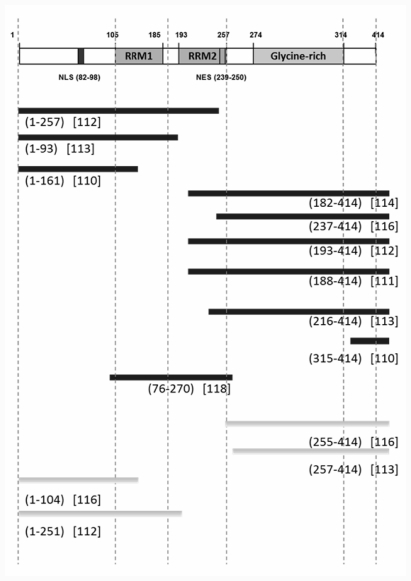
Fragments of TDP-43 and their effects on aggregate propensity or cytotoxicity. Black bars are reported to generate cytosolic aggregates or to induce cell death *in vitro* and *in vivo*. Grey bars were shown not to provide detrimental effects. Amino acid sequence and its source reference are indicated under each bar. References in square brackets are shown in the Reference section.

TDP-43 contains a bipartite lysine- or arginine-rich nuclear translocation signal (NLS) and leucine-rich nuclear export signal (NES), with nuclear-cytosol shuttling regulated by the importin system [105,107]. Substituting the NLS amino acids effectively altered the nuclear localization of TDP-43 [97,108]. In particular, modifying both NLS residues more efficiently induced cytosolic aggregates to be formed than a single amino acid alteration [108]. Studies by Arai *et al*. [83] and Neumann *et al*. [84] identified 35-kDa or 25-kDa fragments in the affected lesions of ALS or FTLD patients. TDP-43 possesses amino acid sequences that mimic caspase recognition sites just carboxyl to the NLS, and the cleavage of these also generated 25-kDa and 35-kDa fragments in the cytosol [109]. Importantly, Nishimoto *et al*. showed TDP-43 fragments of the same molecular sizes produced in the absence of caspase, and that these fragments are also generated as alternate isoforms [110]. This finding suggested that the fragmentation of TDP-43 could be a primary event in triggering ALS, although it remains unclear whether the generation of such aberrant isoforms is a regulated or random event. The cytosolic aggregates were predominantly carboxyl fragments, since they were labeled mostly by anti-*C*-terminal TDP-43, but much less frequently by the antibody recognizing *N*-terminus [111]. Accumulating evidence shows that the *C*-terminus of TDP-43 has a higher propensity for aggregate formation than other domains [112–114]. However, it should be noted that carboxyl fragments without the 2^nd^ RNA binding domain, RRM2 do not form cytosolic aggregates [115]. In agreement with this, recent studies highlighted the importance of RRM2 domains in the cytosolic redistribution or aggregate formation of TDP-43 [114–116], although the molecular basis by which RRM2 would be involved in the TDP-43 misfolding remains unclear. In addition, RRM1, the 1^st^ RNA binding domain, together with the amino terminal fragment of TDP-43 reportedly mediates dimer formation in transfected cells in culture [117]. The possible roles for the various TDP-43 fragments in cytosolic aggregate formation are summarized in Figure 2.

### 3.3. Pathogenic Role of Mislocalized TDP-43 in the Cytosol

The possible role of cytosolic TDP-43 as a trigger for ALS pathology remains unclear. In yeast, TDP-43 fragments without a functional NLS domain redistributed into the cytosol, although only those fragments containing the entire RRM2 and the carboxyl terminus induced toxicity [118]. Moreover, Tg mice expressing TDP-43 with an NLS substitution to alanine showed massive cytosolic aggregates and neuronal death [119]. On the other hand, a study in *C. elegans* revealed the motor phenotype only in the presence of both RRM1 and RRM2 together with the carboxyl terminal, while NLS-defective TDP-43 showed no motor impairment [120]. Aggregate formation of TDP-43 does not immediately follow its cytosolic redistribution, and using the elegant *in vivo* cleavage system with TEV protease, Pesiridis *et al*. [116] found that aggregates containing carboxyl fragments of TDP-43 were actually prompted by the disruption of dynein-mediated transport and RNA interaction. It should also be noted that the cytosolic redistribution of TDP-43 induces nuclear exclusion of the endogenous TDP-43 [108], which may link to the loss of function of nuclear TDP-43. On the other hand, the cytosolic redistribution of TDP-43 does not lead to neurodegeneration in either WT or mutant TDP-43 Tg mice [121,122]. Finally, we found that polyubiquitinated TDP-43 is present to a similar extent between WT and NLS-defective TDP-43, arguing against the early notion that nuclear exclusion triggers the polyubiquitination of TDP-43 [97].

### 3.4. WT and Mutant TDP-43 in Motor Neuron Degeneration

Over 40 mutations involving amino acid replacement have been reported in TDP-43 [123], with most located in the carboxyl terminus (267-414), one mutation (D169G) in RRM1 (103-183), and two (Y214Y, P225P) in RRM2 (190-26) (Table 1). Biochemical studies suggested that mutant TDP-43 has a higher propensity for aggregate formation and carboxyl termini fragmentation of 25- or 35-kDa fragments [124–127]. Moreover, the overexpression of mutant TDP-43 induces cell death in chick embryonic spinal motor neurons [126], primary motor neuron cultures [124], *C. elegans* [128], and transgenic rodents [121,129,130]. Swarup *et al*. [131] found a moderate phenotype expressed by Tg mice driven by the native promoter of human TDP-43, including cognitive impairment and motor coordination with massive cytosolic redistribution of TDP-43, while ALS-linked mutant TDP-43 proteins more readily aggregate in stress granules (SG) than the WT protein [132].

Our review of the literature found limited types of mutations that induce cell death in cultured cells or Tg mice and all of these are located between the carboxyl terminus and the glycine-rich domain (Table 1). Further studies are needed to investigate the effect of mutations in other parts of the TDP-43 protein such as RRM1, RRM2, or the glycine-rich domains. WT TDP-43 transgenic mice have also showed motor neuron degeneration with paralysis [123,131], although several other studies observed no overt phenotype [129,130].

It remains a matter of debate whether TDP-43-induced ALS is caused by a loss or gain of protein function. A sciatic axotomy upregulates TDP-43 in rodents [106], and the expression level of TDP-43 in ALS patients was up to twice that in non-ALS control [131]. It should also be noted that the aberrant cytosolic TDP-43 brings endogenous TDP-43 to the cytosol, inducing loss of TDP-43 function in the nucleus. Further investigation comparing the temporal profiles of TDP-43 expression levels and the mislocalization of TDP-43, at least *in vivo*, would further our understanding of the TDP-43 proteinopathy.

## 4. Aberrant Localization and Therapeutic Hints

Non-cell autonomous neuronal cell death has been implicated in various neurodegenerative diseases [13]. The recent notion that disease-causative proteins can also affect neighboring cells via secretion is attracting significant attention as a possible mechanism underlying the temporal and spatial progression of disease [138,139]. Studies have shown that phosphorylated tau [140], α-synuclein [141], and Huntingtin [142] proteins can spread out and behave like a prion protein in cell culture models of Alzheimer’s, Parkinson’s, and Huntington’s disease, respectively. Moreover, SOD1 aggregates in the culture media were recently detected inside cells, supporting a prion-like behavior of the misfolded SOD1 [143]. Aggregated SOD1 also acquires a seeding effect in which recombinant mutant SOD1 accelerates fibril formation of previously folded SOD1 species [144]. Furthermore, a recent paper has provided evidence that mutant or misfolded human SOD1 induces a species-specific conformational conversion of native WT SOD1 to misfolded species, which is surprisingly mediated by the tryptophan at 32 amino acid residue [145]. We and others proved that extracellular SOD1 mutant can be a therapeutic target for vaccination or antibody therapy against misfolded SOD1 [146–148]. In the case of TDP-43, mounting data supports that TDP-43 aggregates have a seeding effect *in vitro* [114,149], although the possible spreading of TDP-43 is not proven. However, considering the clinical evidence that cerebrospinal fluid TDP-43 [150–152], it is possible that TDP-43 also behaves like a prion. In this sense, if mislocalized TDP-43 triggers loss of function of nuclear TDP-43, elimination of cytosolic TDP-43 is a challenging but attractive therapeutic strategy. For instance, the monoclonal antibody strategy could have extensive applications clinically including using intrabody or Fab molecules for molecular targeting of such species.

## 5. Conclusions

We present evidence in this review that the misdirection of the ALS-linked proteins can ultimately account for various pathological signaling. The efficacy of any molecular targeting strategy depends on our understanding of the aberrant subcellular localization of these proteins. Further investigation is required to uncover the molecular basis and the downstream cascades of the mislocalized, ALS-linked proteins to develop more innovative therapy.

## Figures and Tables

**Table 1:** Previously identified genetic mutations in TDP-43. Mutations in the exon in regard with the cell toxicity are shown.

Domain	Mutation	Cell death	Reference
*N*-terminus	D65E		[133]
	A66A		[133]
	A90V		[126]

RRM1	D169G		[124]

RRM2	Y214Y		[134]
	P225P		[133]
	N267S		[135]

Glycine-rich	G287S		[136]
	G290A		[137]
	G290S		[136]
	G294A		[126]
	G294V		[136]
	G295R		[135]
	G295S		[135]
	G298S		[137]
	M311V		[136]

*C*-terminus	A315A		[133]
	A315T	Tg mice, Cultured cells, Zebrafish	[124,131]
	Q331K	Cultured cells	[126]
	S332N		[135]
	G335D		[136]
	M337V	Tg rat, Cultured cells, Zebrafish, chick embryo	[125,126,129]
	Q343R		[136]
	N345K		[136]
	G348C	Tg mice, Cultured cells, Zebrafish	[124,131]
	N352N		[133]
	N352S		[136]
	[144] 61S		[124]
	P363A		[136]
	Y374X		[136]
	S379C		[136]
	S379P		[135]
	A382P		[136]
	A382T		[124]
	I383V		[136]
	N390D		[124]
	N390S		[136]
	S393L		[135]

## References

[1] Kabashi E, Durham HD (2006). Failure of protein quality control in amyotrophic lateral sclerosis. Biochim. Biophys. Acta.

[2] Hart PJ (2006). Pathogenic superoxide dismutase structure, folding, aggregation and turnover. Curr. Opin. Chem. Biol.

[3] Ticozzi N, Ratti A, Silani V (2010). Protein aggregation and defective RNA metabolism as mechanisms for motor neuron damage. CNS Neurol. Disord. Drug Targets.

[4] Takahashi K, Nakamura H, Okada E (1972). Hereditary amyotrophic lateral sclerosis. Histochemical and electron microscopic study of hyaline inclusions in motor neurons. Arch. Neurol.

[5] Sun CN, Araoz C, Lucas G, Morgan PN, White HJ (1975). Amyotrophic lateral sclerosis. Inclusion bodies in a case of the classic sporadic form. Ann. Clin. Lab. Sci.

[6] Murayama S, Ookawa Y, Mori H, Nakano I, Ihara Y, Kuzuhara S, Tomonaga M (1989). Immunocytochemical and ultrastructural study of Lewy body-like hyaline inclusions in familial amyotrophic lateral sclerosis. Acta Neuropathol. (Berl. ).

[7] Mizusawa H, Nakamura H, Wakayama I, Yen SH, Hirano A (1991). Skein-like inclusions in the anterior horn cells in motor neuron disease. J. Neurol. Sci.

[8] Traub R, Mitsumoto H, Rowland LP (2011). Research advances in amyotrophic lateral sclerosis, 2009 to 2010. Curr. Neurol. Neurosci. Rep.

[9] Maruyama H, Morino H, Ito H, Izumi Y, Kato H, Watanabe Y, Kinoshita Y, Kamada M, Nodera H, Suzuki H (2010). Mutations of optineurin in amyotrophic lateral sclerosis. Nature.

[10] Deng HX, Chen W, Hong ST, Boycott KM, Gorrie GH, Siddique N, Yang Y, Fecto F, Shi Y, Zhai H (2011). Mutations in UBQLN2 cause dominant X-linked juvenile and adult-onset ALS and ALS/dementia. Nature.

[11] Renton AE, Majounie E, Waite A, Simón-Sánchez J, Rollinson S, Gibbs JR, Schymick JC, Laaksovirta H, van Swieten JC, Myllykangas L (2011). A Hexanucleotide repeat expansion in C9ORF72 is the cause of chromosome 9p21-Linked ALS-FTD. Neuron.

[12] Dejesus-Hernandez M, Mackenzie IR, Boeve BF, Boxer AL, Baker M, Rutherford NJ, Nicholson AM, Finch NA, Flynn H, Adamson J (2011). Expanded GGGGCC hexanucleotide repeat in noncoding region of C9ORF72 causes chromosome 9p-Linked FTD and ALS. Neuron.

[13] Ilieva H, Polymenidou M, Cleveland DW (2009). Non-cell autonomous toxicity in neurodegenerative disorders: ALS and beyond. J. Cell Biol.

[14] Bento-Abreu A, Van Damme P, Van Den Bosch L, Robberecht W (2010). The neurobiology of amyotrophic lateral sclerosis. Eur. J. Neurosci.

[15] Kato S, Takikawa M, Nakashima K, Hirano A, Cleveland DW, Kusaka H, Shibata N, Kato M, Nakano I, Ohama E (2000). New consensus research on neuropathological aspects of familial amyotrophic lateral sclerosis with superoxide dismutase 1 (SOD1) gene mutations: Inclusions containing SOD1 in neurons and astrocytes. Amyotroph. Lateral Scler.

[16] Yokoseki A, Shiga A, Tan CF, Tagawa A, Kaneko H, Koyama A, Eguchi H, Tsujino A, Ikeuchi T, Kakita A (2008). TDP-43 mutation in familial amyotrophic lateral sclerosis. Ann. Neurol.

[17] Tateishi T, Hokonohara T, Yamasaki R, Miura S, Kikuchi H, Iwaki A, Tashiro H, Furuya H, Nagara Y, Ohyagi Y (2010). Multiple system degeneration with basophilic inclusions in Japanese ALS patients with FUS mutation. Acta Neuropathol. (Berl. ).

[18] Rosen DR, Siddique T, Patterson D, Figlewicz DA, Sapp P, Hentati A, Donaldson D, Goto J, O’Regan JP, Deng HX (1993). Mutations in Cu/Zn superoxide dismutase gene are associated with familial amyotrophic lateral sclerosis. Nature.

[19] University of Tokyo (2011). ALS Mutation Database.

[20] Reaume AG, Elliott JL, Hoffman EK, Kowall NW, Ferrante RJ, Siwek DF, Wilcox HM, Flood DG, Beal MF, Brown RH (1996). Motor neurons in Cu/Zn superoxide dismutase-deficient mice develop normally but exhibit enhanced cell death after axonal injury. Nat. Genet.

[21] Furukawa Y, Fu R, Deng HX, Siddique T, O’halloran TV (2005). From the Cover: Disulfide cross-linked protein represents a significant fraction of ALS-associated Cu, Zn-superoxide dismutase aggregates in spinal cords of model mice. Proc. Natl. Acad. Sci. USA.

[22] Wang J, Xu G, Borchelt DR (2006). Mapping superoxide dismutase 1 domains of non-native interaction: roles of intra- and intermolecular disulfide bonding in aggregation. J. Neurochem.

[23] Watanabe S, Nagano S, Duce J, Kiaei M, Li QX, Tucker SM, Tiwari A, Brown RH, Beal MF, Hayward LJ (2007). Increased affinity for copper mediated by cysteine 111 in forms of mutant superoxide dismutase 1 linked to amyotrophic lateral sclerosis. Free Radic. Biol. Med.

[24] Fujiwara N, Nakano M, Kato S, Yoshihara D, Ookawara T, Eguchi H, Taniguchi N, Suzuki K (2007). Oxidative modification to cysteine sulfonic Acid of cys111 in human copper-zinc superoxide dismutase. J. Biol. Chem.

[25] Tiwari A, Hayward LJ (2003). Familial amyotrophic lateral sclerosis mutants of copper/zinc superoxide dismutase are susceptible to disulfide reduction. J. Biol. Chem.

[26] Rakhit R, Crow JP, Lepock JR, Kondejewski LH, Cashman NR, Chakrabartty A (2002). Monomeric Cu, Zn-superoxide dismutase is a common misfolding intermediate in the oxidation models of sporadic and familial amyotrophic lateral sclerosis. J. Biol. Chem.

[27] Rakhit R, Robertson J, Vande Velde C, Horne P, Ruth DM, Griffin J, Cleveland DW, Cashman NR, Chakrabartty A (2007). An immunological epitope selective for pathological monomer-misfolded SOD1 in ALS. Nat. Med.

[28] Urushitani M, Kurisu J, Tateno M, Hatakeyama S, Nakayama K, Kato S, Takahashi R (2004). CHIP promotes proteasomal degradation of familial ALS-linked mutant SOD1 by ubiquitinating Hsp/Hsc70. J. Neurochem.

[29] Urushitani M, Kurisu J, Tsukita K, Takahashi R (2002). Proteasomal inhibition by misfolded mutant superoxide dismutase 1 induces selective motor neuron death in familial amyotrophic lateral sclerosis. J. Neurochem.

[30] Urushitani M, Ezzi SA, Matsuo A, Tooyama I, Julien JP (2008). The endoplasmic reticulum-Golgi pathway is a target for translocation and aggregation of mutant superoxide dismutase linked to ALS. FASEB J.

[31] Okado-Matsumoto A, Fridovich I (2002). Amyotrophic lateral sclerosis: a proposed mechanism. Proc. Natl. Acad. Sci. USA.

[32] Shaw BF, Valentine JS (2007). How do ALS-associated mutations in superoxide dismutase 1 promote aggregation of the protein?. Trends Biochem. Sci.

[33] Urushitani M, Nakamizo T, Inoue R, Sawada H, Kihara T, Honda K, Akaike A, Shimohama S (2001). *N*-methyl-d-aspartate receptor-mediated mitochondrial Ca(2+) overload in acute excitotoxic motor neuron death: A mechanism distinct from chronic neurotoxicity after Ca(2+) influx. J. Neurosci. Res.

[34] Wong PC, Pardo CA, Borchelt DR, Lee MK, Copeland NG, Jenkins NA, Sisodia SS, Cleveland DW, Price DL (1995). An adverse property of a familial ALS-linked SOD1 mutation causes motor neuron disease characterized by vacuolar degeneration of mitochondria. Neuron.

[35] Higgins CM, Jung C, Ding H, Xu Z (2002). Mutant Cu, Zn superoxide dismutase that causes motoneuron degeneration is present in mitochondria in the CNS. J Neurosci.

[36] Takeuchi H, Kobayashi Y, Ishigaki S, Doyu M, Sobue G (2002). Mitochondrial localization of mutant superoxide dismutase 1 triggers caspase-dependent cell death in a cellular model of familial amyotrophic lateral sclerosis. J. Biol. Chem.

[37] Inoue H, Tsukita K, Iwasato T, Suzuki Y, Tomioka M, Tateno M, Nagao M, Kawata A, Saido TC, Miura M (2003). The crucial role of caspase-9 in the disease progression of a transgenic ALS mouse model. EMBO J.

[38] Reyes NA, Fisher JK, Austgen K, VandenBerg S, Huang EJ, Oakes SA (2010). Blocking the mitochondrial apoptotic pathway preserves motor neuron viability and function in a mouse model of amyotrophic lateral sclerosis. J. Clin. Invest.

[39] Vande Velde C, McDonald KK, Boukhedimi Y, McAlonis-Downes M, Lobsiger CS, Bel Hadj S, Zandona A, Julien JP, Shah SB, Cleveland DW (2011). Misfolded SOD1 associated with motor neuron mitochondria alters mitochondrial shape and distribution prior to clinical onset. PLoS One.

[40] Israelson A, Arbel N, Da Cruz S, Ilieva H, Yamanaka K, Shoshan-Barmatz V, Cleveland DW (2010). Misfolded mutant SOD1 directly inhibits VDAC1 conductance in a mouse model of inherited ALS. Neuron.

[41] Misawa H, Nakata K, Matsuura J, Moriwaki Y, Kawashima K, Shimizu T, Shirasawa T, Takahashi R (2006). Conditional knockout of Mn superoxide dismutase in postnatal motor neurons reveals resistance to mitochondrial generated superoxide radicals. Neurobiol. Dis.

[42] Zhu YB, Sheng ZH (2011). Increased axonal mitochondrial mobility does not slow amyotrophic lateral sclerosis (ALS)-like disease in mutant SOD1 mice. J. Biol. Chem.

[43] Chang LY, Slot JW, Geuze HJ, Crapo JD (1988). Molecular immunocytochemistry of the CuZn superoxide dismutase in rat hepatocytes. J. Cell Biol.

[44] Saito T, Shinzawa H, Togashi H, Wakabayashi H, Ukai K, Takahashi T, Ishikawa M, Dobashi M, Imai Y (1989). Ultrastructural localization of Cu, Zn-SOD in hepatocytes of patients with various liver diseases. Histol. Histopathol.

[45] Tobisawa S, Hozumi Y, Arawaka S, Koyama S, Wada M, Nagai M, Aoki M, Itoyama Y, Goto K, Kato T (2003). Mutant SOD1 linked to familial amyotrophic lateral sclerosis, but not wild-type SOD1, induces ER stress in COS7 cells and transgenic mice. Biochem. Biophys. Res. Commun.

[46] Urushitani M, Sik A, Sakurai T, Nukina N, Takahashi R, Julien JP (2006). Chromogranin-mediated secretion of mutant superoxide dismutase proteins linked to amyotrophic lateral sclerosis. Nat. Neurosci.

[47] Kikuchi H, Almer G, Yamashita S, Guegan C, Nagai M, Xu Z, Sosunov AA, McKhann GM, Przedborski S (2006). Spinal cord endoplasmic reticulum stress associated with a microsomal accumulation of mutant superoxide dismutase-1 in an ALS model. Proc. Natl. Acad. Sci USA.

[48] Mori A, Yamashita S, Uchino K, Suga T, Ikeda T, Takamatsu K, Ishizaki M, Koide T, Kimura E, Mita S (2011). Derlin-1 overexpression ameliorates mutant SOD1-induced endoplasmic reticulum stress by reducing mutant SOD1 accumulation. Neurochem. Int.

[49] Atkin JD, Farg MA, Walker AK, McLean C, Tomas D, Horne MK (2008). Endoplasmic reticulum stress and induction of the unfolded protein response in human sporadic amyotrophic lateral sclerosis. Neurobiol. Dis.

[50] Walker AK, Atkin JD (2011). Stress signaling from the endoplasmic reticulum: A central player in the pathogenesis of amyotrophic lateral sclerosis. IUBMB Life.

[51] Sasaki S (2010). Endoplasmic reticulum stress in motor neurons of the spinal cord in sporadic amyotrophic lateral sclerosis. J. Neuropathol. Exp. Neurol.

[52] Ito Y, Yamada M, Tanaka H, Aida K, Tsuruma K, Shimazawa M, Hozumi I, Inuzuka T, Takahashi H, Hara H (2009). Involvement of CHOP, an ER-stress apoptotic mediator, in both human sporadic ALS and ALS model mice. Neurobiol. Dis.

[53] Saxena S, Cabuy E, Caroni P (2009). A role for motoneuron subtype-selective ER stress in disease manifestations of FALS mice. Nat. Neurosci.

[54] Nagata T, Ilieva H, Murakami T, Shiote M, Narai H, Ohta Y, Hayashi T, Shoji M, Abe K (2007). Increased ER stress during motor neuron degeneration in a transgenic mouse model of amyotrophic lateral sclerosis. Neurol. Res.

[55] Kieran D, Woods I, Villunger A, Strasser A, Prehn JH (2007). Deletion of the BH3-only protein puma protects motoneurons from ER stress-induced apoptosis and delays motoneuron loss in ALS mice. Proc. Natl. Acad. Sci. USA.

[56] Nishitoh H, Kadowaki H, Nagai A, Maruyama T, Yokota T, Fukutomi H, Noguchi T, Matsuzawa A, Takeda K, Ichijo H (2008). ALS-linked mutant SOD1 induces ER stress- and ASK1-dependent motor neuron death by targeting Derlin-1. Genes Dev.

[57] Atkin JD, Farg MA, Turner BJ, Tomas D, Lysaght JA, Nunan J, Rembach A, Nagley P, Beart PM, Cheema SS (2006). Induction of the unfolded protein response in familial amyotrophic lateral sclerosis and association of protein-disulfide isomerase with superoxide dismutase 1. J. Biol. Chem.

[58] Turner BJ, Atkin JD (2006). ER stress and UPR in familial amyotrophic lateral sclerosis. Curr. Mol. Med.

[59] Mourelatos Z, Adler H, Hirano A, Donnenfeld H, Gonatas JO, Gonatas NK (1990). Fragmentation of the Golgi apparatus of motor neurons in amyotrophic lateral sclerosis revealed by organelle-specific antibodies. Proc. Natl. Acad. Sci. USA.

[60] Lafon-Cazal M, Adjali O, Galéotti N, Poncet J, Jouin P, Homburger V, Bockaert J, Marin P (2003). Proteomic analysis of astrocytic secretion in the mouse. Comparison with the cerebrospinal fluid proteome. J. Biol. Chem.

[61] Turner BJ, Atkin JD, Farg MA, Zang da W, Rembach A, Lopes EC, Patch JD, Hill AF, Cheema SS (2005). Impaired extracellular secretion of mutant superoxide dismutase 1 associates with neurotoxicity in familial amyotrophic lateral sclerosis. J. Neurosci.

[62] Durham HD, Roy J, Dong L, Figlewicz DA (1997). Aggregation of mutant Cu/Zn superoxide dismutase proteins in a culture model of ALS. J. Neuropathol. Exp. Neurol.

[63] Matsumoto G, Stojanovic A, Holmberg CI, Kim S, Morimoto RI (2005). Structural properties and neuronal toxicity of amyotrophic lateral sclerosis-associated Cu/Zn superoxide dismutase 1 aggregates. J. Cell Biol.

[64] Pramatarova A, Laganière J, Roussel J, Brisebois K, Rouleau GA (2001). Neuron-specific expression of mutant superoxide dismutase 1 in transgenic mice does not lead to motor impairment. J. Neurosci.

[65] Wang LJ, Lu YY, Muramatsu S, Ikeguchi K, Fujimoto K, Okada T, Mizukami H, Matsushita T, Hanazono Y, Kume A (2002). Neuroprotective effects of glial cell line-derived neurotrophic factor mediated by an adeno-associated virus vector in a transgenic animal model of amyotrophic lateral sclerosis. J. Neurosci.

[66] Gong YH, Parsadanian AS, Andreeva A, Snider WD, Elliott JL (2000). Restricted expression of G86R Cu/Zn superoxide dismutase in astrocytes results in astrocytosis but does not cause motoneuron degeneration. J. Neurosci.

[67] Clement AM, Nguyen MD, Roberts EA, Garcia ML, Boillée S, Rule M, McMahon AP, Doucette W, Siwek D, Ferrante RJ (2003). Wild-type nonneuronal cells extend survival of SOD1 mutant motor neurons in ALS mice. Science.

[68] Yamanaka K, Chun SJ, Boillee S, Fujimori-Tonou N, Yamashita H, Gutmann DH, Takahashi R, Misawa H, Cleveland DW (2008). Astrocytes as determinants of disease progression in inherited amyotrophic lateral sclerosis. Nat. Neurosci.

[69] Wang L, Gutmann DH, Roos RP (2011). Astrocyte loss of mutant SOD1 delays ALS disease onset and progression in G85R transgenic mice. Hum. Mol. Genet.

[70] Wang L, Sharma K, Deng HX, Siddique T, Grisotti G, Liu E, Roos RP (2008). Restricted expression of mutant SOD1 in spinal motor neurons and interneurons induces motor neuron pathology. Neurobiol. Dis.

[71] Nagai M, Re DB, Nagata T, Chalazonitis A, Jessell TM, Wichterle H, Przedborski S (2007). Astrocytes expressing ALS-linked mutated SOD1 release factors selectively toxic to motor neurons. Nat. Neurosci.

[72] Di Giorgio FP, Carrasco MA, Siao MC, Maniatis T, Eggan K (2007). Non-cell autonomous effect of glia on motor neurons in an embryonic stem cell-based ALS model. Nat. Neurosci.

[73] Di Giorgio FP, Boulting GL, Bobrowicz S, Eggan KC (2008). Human embryonic stem cell-derived motor neurons are sensitive to the toxic effect of glial cells carrying an ALS-causing mutation. Cell Stem Cell.

[74] Zhao W, Xie W, Xiao Q, Beers DR, Appel SH (2006). Protective effects of an anti-inflammatory cytokine, interleukin-4, on motoneuron toxicity induced by activated microglia. J. Neurochem.

[75] Zhao W, Beers DR, Henkel JS, Zhang W, Urushitani M, Julien JP, Appel SH (2010). Extracellular mutant SOD1 induces microglial-mediated motoneuron injury. Glia.

[76] Ezzi SA, Larivière R, Urushitani M, Julien JP (2010). Neuronal over-expression of chromogranin A accelerates disease onset in a mouse model of ALS. J. Neurochem.

[77] Liu HN, Sanelli T, Horne P, Pioro EP, Strong MJ, Rogaeva E, Bilbao J, Zinman L, Robertson J (2009). Lack of evidence of monomer/misfolded superoxide dismutase-1 in sporadic amyotrophic lateral sclerosis. Ann. Neurol.

[78] Zetterström P, Andersen PM, Brännström T, Marklund SL (2011). Misfolded superoxide dismutase-1 in CSF from amyotrophic lateral sclerosis patients. J. Neurochem.

[79] Ezzi SA, Urushitani M, Julien JP (2007). Wild-type superoxide dismutase acquires binding and toxic properties of ALS-linked mutant forms through oxidation. J. Neurochem.

[80] Gruzman A, Wood WL, Alpert E, Prasad MD, Miller RG, Rothstein JD, Bowser R, Hamilton R, Wood TD, Cleveland DW (2007). Common molecular signature in SOD1 for both sporadic and familial amyotrophic lateral sclerosis. Proc. Natl. Acad. Sci. USA.

[81] Bosco DA, Morfini G, Karabacak NM, Song Y, Gros-Louis F, Pasinelli P, Goolsby H, Fontaine BA, Lemay N, McKenna-Yasek D (2010). Wild-type and mutant SOD1 share an aberrant conformation and a common pathogenic pathway in ALS. Nat. Neurosci.

[82] Haidet-Phillips AM, Hester ME, Miranda CJ, Meyer K, Braun L, Frakes A, Song S, Likhite S, Murtha MJ, Foust KD (2011). Astrocytes from familial and sporadic ALS patients are toxic to motor neurons. Nat. Biotechnol.

[83] Arai T, Hasegawa M, Akiyama H, Ikeda K, Nonaka T, Mori H, Mann D, Tsuchiya K, Yoshida M, Hashizume Y (2006). TDP-43 is a component of ubiquitin-positive tau-negative inclusions in frontotemporal lobar degeneration and amyotrophic lateral sclerosis. Biochem. Biophys. Res. Commun.

[84] Neumann M, Sampathu DM, Kwong LK, Truax AC, Micsenyi MC, Chou TT, Bruce J, Schuck T, Grossman M, Clark CM (2006). Ubiquitinated TDP-43 in frontotemporal lobar degeneration and amyotrophic lateral sclerosis. Science.

[85] Borroni B, Bonvicini C, Alberici A, Buratti E, Agosti C, Archetti S, Papetti A, Stuani C, Di Luca M, Gennarelli M (2009). Mutation within TARDBP leads to frontotemporal dementia without motor neuron disease. Hum. Mutat.

[86] Benajiba L, Le Ber I, Camuzat A, Lacoste M, Thomas-Anterion C, Couratier P, Legallic S, Salachas F, Hannequin D, Decousus M (2009). TARDBP mutations in motoneuron disease with frontotemporal lobar degeneration. Ann. Neurol.

[87] Quadri M, Cossu G, Saddi V, Simons EJ, Murgia D, Melis M, Ticca A, Oostra BA, Bonifati V (2011). Broadening the phenotype of TARDBP mutations: The TARDBP Ala382Thr mutation and Parkinson’s disease in Sardinia. Neurogenetics.

[88] Buratti E, Dork T, Zuccato E, Pagani F, Romano M, Baralle FE (2001). Nuclear factor TDP-43 and SR proteins promote *in vitro* and *in vivo* CFTR exon 9 skipping. EMBO J.

[89] Strong MJ, Volkening K, Hammond R, Yang W, Strong W, Leystra-Lantz C, Shoesmith C (2007). TDP43 is a human low molecular weight neurofilament (hNFL) mRNA-binding protein. Mol. Cell. Neurosci.

[90] Rogelj B, Briese M, Cereda M, Kayikci M, König J, Hortobágyi T, Nishimura AL, Zupunski V, Patani R, Chandran S (2011). Characterizing the RNA targets and position-dependent splicing regulation by TDP-43. Nat. Neurosci.

[91] Polymenidou M, Lagier-Tourenne C, Hutt KR, Huelga SC, Moran J, Liang TY, Ling SC, Sun E, Wancewicz E, Mazur C (2011). Long pre-mRNA depletion and RNA missplicing contribute to neuronal vulnerability from loss of TDP-43. Nat. Neurosci.

[92] Xiao S, Sanelli T, Dib S, Sheps D, Findlater J, Bilbao J, Keith J, Zinman L, Rogaeva E, Robertson J (2011). RNA targets of TDP-43 identified by UV-CLIP are deregulated in ALS. Mol. Cell. Neurosci.

[93] Ayala YM, Misteli T, Baralle FE (2008). TDP-43 regulates retinoblastoma protein phosphorylation through the repression of cyclin-dependent kinase 6 expression. Proc. Natl. Acad. Sci. USA.

[94] Fiesel FC, Voigt A, Weber SS, Van den Haute C, Waldenmaier A, Görner K, Walter M, Anderson ML, Kern JV, Rasse TM (2010). Knockdown of transactive response DNA-binding protein (TDP-43) downregulates histone deacetylase 6. EMBO J.

[95] Iguchi Y, Katsuno M, Niwa J, Yamada S, Sone J, Waza M, Adachi H, Tanaka F, Nagata K, Arimura N (2009). TDP-43 depletion induces neuronal cell damage through dysregulation of Rho family GTPases. J. Biol. Chem.

[96] Anthony K, Gallo JM (2010). Aberrant RNA processing events in neurological disorders. Brain Res.

[97] Urushitani M, Sato T, Bamba H, Hisa Y, Tooyama I (2010). Synergistic effect between proteasome and autophagosome in the clearance of polyubiquitinated TDP-43. J. Neurosci. Res.

[98] Mackenzie IR, Bigio EH, Ince PG, Geser F, Neumann M, Cairns NJ, Kwong LK, Forman MS, Ravits J, Stewart H (2007). Pathological TDP-43 distinguishes sporadic amyotrophic lateral sclerosis from amyotrophic lateral sclerosis with SOD1 mutations. Ann. Neurol.

[99] Sumi H, Kato S, Mochimaru Y, Fujimura H, Etoh M, Sakoda S (2009). Nuclear TAR DNA binding protein 43 expression in spinal cord neurons correlates with the clinical course in amyotrophic lateral sclerosis. J. Neuropathol. Exp. Neurol.

[100] Okamoto Y, Ihara M, Urushitani M, Yamashita H, Kondo T, Tanigaki A, Oono M, Komatsu K, Kawamata J, Ikemoto A (2011). An autopsy case of SOD1-related ALS with TDP-43 positive inclusions. Neurology.

[101] Wider C, Dickson DW, Stoessl AJ, Tsuboi Y, Chapon F, Gutmann L, Lechevalier B, Calne DB, Personett DA, Hulihan M (2009). Pallidonigral TDP-43 pathology in Perry syndrome. Parkinsonism Relat. Disord.

[102] Nakashima-Yasuda H, Uryu K, Robinson J, Xie SX, Hurtig H, Duda JE, Arnold SE, Siderowf A, Grossman M, Leverenz JB (2007). Co-morbidity of TDP-43 proteinopathy in Lewy body related diseases. Acta Neuropathol. (Berl. ).

[103] Schwab C, Arai T, Hasegawa M, Yu S, McGeer PL (2008). Colocalization of transactivation-responsive DNA-binding protein 43 and huntingtin in inclusions of Huntington disease. J. Neuropathol. Exp. Neurol.

[104] Weihl CC, Temiz P, Miller SE, Watts G, Smith C, Forman M, Hanson PI, Kimonis V, Pestronk A (2008). TDP-43 accumulation in inclusion body myopathy muscle suggests a common pathogenic mechanism with frontotemporal dementia. J. Neurol. Neurosurg. Psychiatry.

[105] Sato T, Takeuchi S, Saito A, Ding W, Bamba H, Matsuura H, Hisa Y, Tooyama I, Urushitani M (2009). Axonal ligation induces transient redistribution of TDP-43 in brainstem motor neurons. Neuroscience.

[106] Moisse K, Volkening K, Leystra-Lantz C, Welch I, Hill T, Strong MJ (2009). Divergent patterns of cytosolic TDP-43 and neuronal progranulin expression following axotomy: Implications for TDP-43 in the physiological response to neuronal injury. Brain Res.

[107] Nishimura AL, Zupunski V, Troakes C, Kathe C, Fratta P, Howell M, Gallo JM, Hortobágyi T, Shaw CE, Rogelj B (2010). Nuclear import impairment causes cytoplasmic trans-activation response DNA-binding protein accumulation and is associated with frontotemporal lobar degeneration. Brain.

[108] Winton MJ, Igaz LM, Wong MM, Kwong LK, Trojanowski JQ, Lee VM (2008). Disturbance of nuclear and cytoplasmic Tar DNA binding protein (TDP-43) induces disease-like redistribution, sequestration and aggregate formation. J. Biol. Chem.

[109] Zhang YJ, Xu YF, Dickey CA, Buratti E, Baralle F, Bailey R, Pickering-Brown S, Dickson D, Petrucelli L (2007). Progranulin mediates caspase-dependent cleavage of TAR DNA binding protein-43. J. Neurosci.

[110] Nishimoto Y, Ito D, Yagi T, Nihei Y, Tsunoda Y, Suzuki N (2010). Characterization of alternative isoforms and inclusion body of the TAR DNA-binding protein-43. J. Biol. Chem.

[111] Igaz LM, Kwong LK, Xu Y, Truax AC, Uryu K, Neumann M, Clark CM, Elman LB, Miller BL, Grossman M (2008). Enrichment of C-Terminal fragments in TAR DNA-Binding Protein-43 cytoplasmic inclusions in brain but not in spinal cord of frontotemporal lobar degeneration and amyotrophic lateral sclerosis. Am. J. Pathol.

[112] Nonaka T, Kametani F, Arai T, Akiyama H, Hasegawa M (2009). Truncation and pathogenic mutations facilitate the formation of intracellular aggregates of TDP-43. Hum. Mol. Genet.

[113] Johnson BS, Snead D, Lee JJ, McCaffery JM, Shorter J, Gitler AD (2009). TDP-43 is intrinsically aggregation-prone, and amyotrophic lateral sclerosis-linked mutations accelerate aggregation and increase toxicity. J. Biol. Chem.

[114] Furukawa Y, Kaneko K, Watanabe S, Yamanaka K, Nukina N (2011). A seeding reaction recapitulates intracellular formation of Sarkosyl-insoluble transactivation response element (TAR) DNA-binding protein-43 inclusions. J. Biol. Chem.

[115] Yang C, Tan W, Whittle C, Qiu L, Cao L, Akbarian S, Xu Z (2010). The *C*-Terminal TDP-43 fragments have a high aggregation propensity and harm neurons by a dominant-negative mechanism. PLoS One.

[116] Pesiridis GS, Tripathy K, Tanik S, Trojanowski JQ, Lee VM (2011). A “two-hit” hypothesis for inclusion formation by carboxyl-terminal fragments of TDP-43 protein linked to RNA depletion and impaired microtubule-dependent transport. J. Biol. Chem.

[117] Shiina Y, Arima K, Tabunoki H, Satoh J (2010). TDP-43 dimerizes in human cells in culture. Cell. Mol. Neurobiol.

[118] Johnson BS, McCaffery JM, Lindquist S, Gitler AD (2008). A yeast TDP-43 proteinopathy model: Exploring the molecular determinants of TDP-43 aggregation and cellular toxicity. Proc. Natl. Acad. Sci. USA.

[119] Igaz LM, Kwong LK, Lee EB, Chen-Plotkin A, Swanson E, Unger T, Malunda J, Xu Y, Winton MJ, Trojanowski JQ (2011). Dysregulation of the ALS-associated gene TDP-43 leads to neuronal death and degeneration in mice. J. Clin. Invest.

[120] Ash PE, Zhang YJ, Roberts CM, Saldi T, Hutter H, Buratti E, Petrucelli L, Link CD (2010). Neurotoxic effects of TDP-43 overexpression in *C. elegans*. Hum. Mol. Genet.

[121] Wegorzewska I, Bell S, Cairns NJ, Miller TM, Baloh RH (2009). TDP-43 mutant transgenic mice develop features of ALS and frontotemporal lobar degeneration. Proc. Natl. Acad. Sci. USA.

[122] Xu YF, Gendron TF, Zhang YJ, Lin WL, D’Alton S, Sheng H, Casey MC, Tong J, Knight J, Yu X (2010). Wild-type human TDP-43 expression causes TDP-43 phosphorylation, mitochondrial aggregation, motor deficits, and early mortality in transgenic mice. J. Neurosci.

[123] University of Tokyo (2011). ALS Mutation Database.

[124] Kabashi E, Valdmanis PN, Dion P, Spiegelman D, McConkey BJ, Velde CV, Bouchard JP, Lacomblez L, Pochigaeva K, Salachas F (2008). TARDBP mutations in individuals with sporadic and familial amyotrophic lateral sclerosis. Nat. Genet.

[125] Kabashi E, Lin L, Tradewell ML, Dion PA, Bercier V, Bourgouin P, Rochefort D, Bel Hadj S, Durham HD, Vande Velde C (2010). Gain and loss of function of ALS-related mutations of TARDBP (TDP-43) cause motor deficits *in vivo*. Hum. Mol. Genet.

[126] Sreedharan J, Blair IP, Tripathi VB, Hu X, Vance C, Rogelj B, Ackerley S, Durnall JC, Williams KL, Buratti E (2008). TDP-43 mutations in familial and sporadic amyotrophic lateral sclerosis. Science.

[127] Rutherford NJ, Zhang YJ, Baker M, Gass JM, Finch NA, Xu YF, Stewart H, Kelley BJ, Kuntz K, Crook RJ (2008). Novel mutations in TARDBP (TDP-43) in patients with familial amyotrophic lateral sclerosis. PLoS Genet.

[128] Liachko NF, Guthrie CR, Kraemer BC (2010). Phosphorylation promotes neurotoxicity in a Caenorhabditis elegans model of TDP-43 proteinopathy. J. Neurosci.

[129] Zhou H, Huang C, Chen H, Wang D, Landel CP, Xia PY, Bowser R, Liu YJ, Xia XG (2010). Transgenic rat model of neurodegeneration caused by mutation in the TDP gene. PLoS Genet.

[130] Stallings NR, Puttaparthi K, Luther CM, Burns DK, Elliott JL (2010). Progressive motor weakness in transgenic mice expressing human TDP-43. Neurobiol. Dis.

[131] Swarup V, Phaneuf D, Bareil C, Robertson J, Rouleau GA, Kriz J, Julien JP (2011). Pathological hallmarks of amyotrophic lateral sclerosis/frontotemporal lobar degeneration in transgenic mice produced with TDP-43 genomic fragments. Brain.

[132] Liu-Yesucevitz L, Bilgutay A, Zhang YJ, Vanderwyde T, Citro A, Mehta T, Zaarur N, McKee A, Bowser R, Sherman M (2010). Tar DNA binding protein-43 (TDP-43) associates with stress granules: Analysis of cultured cells and pathological brain tissue. PLoS One.

[133] Guerreiro RJ, Schymick JC, Crews C, Singleton A, Hardy J, Traynor BJ (2008). TDP-43 is not a common cause of sporadic amyotrophic lateral sclerosis. PLoS One.

[134] Gijselinck I, Sleegers K, Engelborghs S, Robberecht W, Martin JJ, Vandenberghe R, Sciot R, Dermaut B, Goossens D, van der Zee J (2009). Neuronal inclusion protein TDP-43 has no primary genetic role in FTD and ALS. Neurobiol. Aging.

[135] Corrado L, Ratti A, Gellera C, Buratti E, Castellotti B, Carlomagno Y, Ticozzi N, Mazzini L, Testa L, Taroni F (2009). High frequency of TARDBP gene mutations in Italian patients with amyotrophic lateral sclerosis. Hum. Mutat.

[136] Lagier-Tourenne C, Cleveland DW (2009). Rethinking ALS: the FUS about TDP-43. Cell.

[137] Van Deerlin VM, Leverenz JB, Bekris LM, Bird TD, Yuan W, Elman LB, Clay D, Wood EM, Chen-Plotkin AS, Martinez-Lage M (2008). TARDBP mutations in amyotrophic lateral sclerosis with TDP-43 neuropathology: A genetic and histopathological analysis. Lancet. Neurol.

[138] Aguzzi A, Rajendran L (2009). The transcellular spread of cytosolic amyloids, prions, and prionoids. Neuron.

[139] Frost B, Diamond MI (2010). Prion-like mechanisms in neurodegenerative diseases. Nat. Rev. Neurosci.

[140] Frost B, Ollesch J, Wille H, Diamond MI (2009). Conformational diversity of wild-type Tau fibrils specified by templated conformation change. J. Biol. Chem.

[141] Desplats P, Lee HJ, Bae EJ, Patrick C, Rockenstein E, Crews L, Spencer B, Masliah E, Lee SJ (2009). Inclusion formation and neuronal cell death through neuron-to-neuron transmission of alpha-synuclein. Proc. Natl. Acad. Sci. USA.

[142] Ren PH, Lauckner JE, Kachirskaia I, Heuser JE, Melki R, Kopito RR (2009). Cytoplasmic penetration and persistent infection of mammalian cells by polyglutamine aggregates. Nat. Cell Biol.

[143] Münch C, O’Brien J, Bertolotti A (2011). Prion-like propagation of mutant superoxide dismutase-1 misfolding in neuronal cells. Proc. Natl. Acad. Sci. USA.

[144] Chia R, Tattum MH, Jones S, Collinge J, Fisher EM, Jackson GS (2010). Superoxide dismutase 1 and tgSOD1 mouse spinal cord seed fibrils, suggesting a propagative cell death mechanism in amyotrophic lateral sclerosis. PLoS One.

[145] Grad LI, Guest WC, Yanai A, Pokrishevsky E, O’Neill MA, Gibbs E, Semenchenko V, Yousefi M, Wishart DS, Plotkin SS (2011). Intermolecular transmission of superoxide dismutase 1 misfolding in living cells. Proc. Natl. Acad. Sci. USA.

[146] Urushitani M, Ezzi SA, Julien JP (2007). Therapeutic effects of immunization with mutant superoxide dismutase in mice models of amyotrophic lateral sclerosis. Proc. Natl. Acad. Sci. USA.

[147] Takeuchi S, Fujiwara N, Ido A, Oono M, Takeuchi Y, Tateno M, Suzuki K, Takahashi R, Tooyama I, Taniguchi N (2010). Induction of protective immunity by vaccination with wild-type apo superoxide dismutase 1 in mutant SOD1 transgenic mice. J. Neuropathol. Exp. Neurol.

[148] Gros-Louis F, Soucy G, Larivière R, Julien JP (2010). Intracerebroventricular infusion of monoclonal antibody or its derived Fab fragment against misfolded forms of SOD1 mutant delays mortality in a mouse model of ALS. J. Neurochem.

[149] Che MX, Jiang YJ, Xie YY, Jiang LL, Hu HY (2011). Aggregation of the 35-kDa fragment of TDP-43 causes formation of cytoplasmic inclusions and alteration of RNA processing. FASEB J.

[150] Noto Y, Shibuya K, Sato Y, Kanai K, Misawa S, Sawai S, Mori M, Uchiyama T, Isose S, Nasu S (2011). Elevated CSF TDP-43 levels in amyotrophic lateral sclerosis: Specificity, sensitivity, and a possible prognostic value. Amyotroph. Lateral Scler.

[151] Steinacker P, Hendrich C, Sperfeld AD, Jesse S, von Arnim CA, Lehnert S, Pabst A, Uttner I, Tumani H, Lee VM (2008). TDP-43 in cerebrospinal fluid of patients with frontotemporal lobar degeneration and amyotrophic lateral sclerosis. Arch. Neurol.

[152] Kasai T, Tokuda T, Ishigami N, Sasayama H, Foulds P, Mitchell DJ, Mann DM, Allsop D, Nakagawa M (2009). Increased TDP-43 protein in cerebrospinal fluid of patients with amyotrophic lateral sclerosis. Acta Neuropathol. (Berl. ).

